# Relationship of *ALDH2* rs671 and *CYP2E1* rs2031920 with hepatocellular carcinoma susceptibility in East Asians: a meta-analysis

**DOI:** 10.1186/s12957-020-1796-0

**Published:** 2020-01-27

**Authors:** Junhong Chen, Weicong Pan, Yongjin Chen, Lijia Wen, Jihao Tu, Kai Liu

**Affiliations:** 1 College of Clinical Medicine, Jilin University, Changchun, China; 2 Department of Hepatopancreatobiliary Surgery, The First Hospital, Jilin University, Changchun, China

**Keywords:** ALDH2, CYP2E1, Hepatocellular carcinoma, Polymorphism

## Abstract

**Background:**

Aldehyde dehydrogenase 2 (ALDH2) and cytochrome p450 2E1 (CYP2E1) are important alcohol-metabolizing enzymes. The aim of this meta-analysis was to evaluate the association of *ALDH2* rs671 and *CYP2E1* rs2031920 polymorphisms with hepatocellular carcinoma (HCC) susceptibility in East Asians.

**Methods:**

A systematic search strategy was implemented in MEDLINE, PubMed, Scopus, Embase, and China Academic Journals databases. Nineteen case-control studies were selected for inclusion. Pooled odds ratios (ORs) and 95% confidence intervals (CIs) were calculated through random-effects or fixed-effects models. Subgroup analysis, meta-regression, sensitivity analysis, cumulative meta-analysis, and evaluation of publication bias were performed.

**Results:**

The overall meta-analysis did not find a significant association of *ALDH2* rs671 and *CYP2E1* rs2031920 genotypes with HCC susceptibility in East Asians. In addition, stratified analysis by country, Hardy-Weinberg equilibrium status, and source of controls also did not identify any association.

**Conclusion:**

The *ALDH2* rs671 and *CYP2E1* rs2031920 polymorphisms are not associated with HCC susceptibility in East Asians.

## Introduction

Hepatocellular carcinoma (HCC) is the most common primary liver cancer and is the third most common cause of cancer-related death. In sub-Saharan Africa and some parts of Asia, it is the leading cause of cancer death. HCC most commonly develops in chronic liver disease patients, the etiology of which includes hepatitis B virus (HBV) and hepatitis C virus (HCV) infection, alcohol, aflatoxin exposure, hemachromatosis, and α1-antitrypsin deficiency [1]. It is likely that HCC arises as a consequence of complex interactions between genetic risk factors and environmental exposures. Candidate gene and genome-wide association studies have started to explore this area, but the role of genetic factors in HCC development remains poorly understood.

Aldehyde dehydrogenase 2 (ALDH2) is a mitochondrial enzyme, which is known for its role in alcohol detoxification. It has the highest affinity for acetaldehyde (ACE) and mediates the rate-limiting step of metabolizing ACE to acetic acid. In addition, ALDH2 metabolizes other aldehydes generated during oxidative stress such as 4-hydroxy-2-nonenal (4-HNE), protecting against oxidative stress [2]. In the human *ALDH2* gene, there is a G-to-A point mutation at exon 12, resulting in a glutamic acid-to-lysine substitution at residue 487 (rs671, Glu>Lys) of the ALDH2 protein (designated ALDH2*2) [3]. The rs671 polymorphism is found in nearly 35–50% of East Asian populations but has not been found in Africans or Caucasians [4]. It is associated with a reduction in the ALDH2 enzymatic activity by 70 and 98% in heterozygotes and homozygotes, respectively [5]. There are multiple association studies assessing the relationship between *ALDH2* rs671 and HCC risk in East Asians. The study by Takeshita et al. was the first study to evaluate the association of *ALDH2* rs671 with HCC susceptibility, finding no association of the *ALDH2* genotypes with HCC development [6]. Their results were supported by several other studies including the study by Liu et al. which was based on a large sample size (600 cases and 3221 controls) [7]. However, the study by Sakamoto et al. suggested that *ALDH2* rs671 might modify the risk for developing HCC [8]. The discrepancies among these studies may be due to the modest effect of the polymorphism, variation in ethnic background, and different sample sizes these studies used. Because the findings remain controversial, a quantitative analysis is needed to assess the evidence.

Cytochrome p450 2E1 (CYP2E1) is also one of the important alcohol-metabolizing enzymes. It is strongly expressed in the liver but can also be found in extrahepatic organs such as the brain and kidneys [9]. Hepatic CYP2E1 levels can be induced by chronic alcohol consumption. CYP2E1 metabolizes ethanol and numerous chemicals including environmental pollutants and clinical drugs. Its highly uncoupled NADPH oxidase activity generates high levels of reactive oxygen species, leading to hepatic lipid peroxidation, cell stress, and apoptosis [10]. Human *CYP2E1* is located on chromosome 10q26.3 and consists of nine exons and eight introns. It is shown that a restriction fragment length polymorphism (rs2031920, Pst I/Rsa) in the 5′-transcriptional region may modify the CYP2E1 enzyme function or mRNA expression levels [11]. Although several studies from East Asia evaluated the possible association of rs2031920 with HCC susceptibility, the results have been conflicting.

In this study, we aim to perform a meta-analysis to assess the relationship of *ALDH2* rs671 and *CYP2E1* rs2031920 with HCC susceptibility in East Asian populations.

## Methods

### Databases and search strategy

Searches were performed in MEDLINE, PubMed, Scopus, Embase, and China Academic Journals databases from inception to July 8, 2019, by two independent authors (Additional file 1). Searches were built around the keywords: “hepatocellular carcinoma,” “liver cancer,” “aldehyde dehydrogenase 2,” “ALDH2,” “cytochrome p450 2E1,” “CYP2E1,” “polymorphism,” “genetic variant,” “susceptibility,” and “development.” No restrictions on language or setting were applied. Titles and abstracts were screened against the inclusion and exclusion criteria. Full texts of potentially eligible studies were screened. Reference lists of all included studies and relevant reviews were hand-searched to identify additional eligible studies. The design and report of our meta-analysis followed the Preferred Reporting Items for Systematic Reviews and Meta-Analyses (PRISMA) guidelines [12].

### Inclusion and exclusion criteria

After the removal of duplicates from different databases, the titles and abstracts of the citations were carefully screened. Irrelevant papers were excluded, leaving potential studies for further full-text evaluation. The inclusion and exclusion criteria for the studies were as follows: (1) case-control studies of unrelated individuals using a population or hospital-based design, (2) evaluation of the relation of *ALDH2* and *CYP2E1* polymorphisms with susceptibility to HCC, and (3) sufficient data for pooling the odds ratio (OR) and 95% confidence interval (CI). Exclusion criteria were studies in languages other than English and Chinese, review articles, meeting abstracts, editorials, and animal studies.

### Data extraction and quality assessment

Two authors extracted data from the eligible studies using a standardized template. Data were collected on first author, country of study, year, number of HCC patients and controls, demographics of HCC patients, source of controls, matching criteria, genotyping methods, and counts of genotypes and alleles (*ALDH2* rs671 and *CYP2E1* rs2031920). The quality of the included studies was evaluated according to the Newcastle Ottawa Scale (NOS) (www.ohri.ca/programs/clinical_epidemiology/oxford.asp).

### Data analysis

Since all meta-analyses conducted involved the use of dichotomous data, summary OR with 95% CIs were presented as the effect measure. The minor allele was considered the at-risk allele. ORs were pooled according to fixed- or random-effects models. The analyses were stratified according to country, Hardy-Weinberg equilibrium status, and source of controls. Heterogeneity was evaluated using the *I*^2^ statistic, with values higher than 50% indicating substantial heterogeneity [13]. We performed a sensitivity analysis to ensure that the effect sizes of our meta-analysis were not driven by any one study. We used sensitivity analysis, meta-regression, and Galbraith plot to identify the main contributors to between-study heterogeneity. A cumulative meta-analysis was performed to explore the trend in the effect sizes. Egger’s test and funnel plots were applied to assess publication bias. All statistical analyses were conducted using STATA 13.0 (Stata, College Station, TX, USA).

## Results

### Summary of included studies

**Fig. 1:**
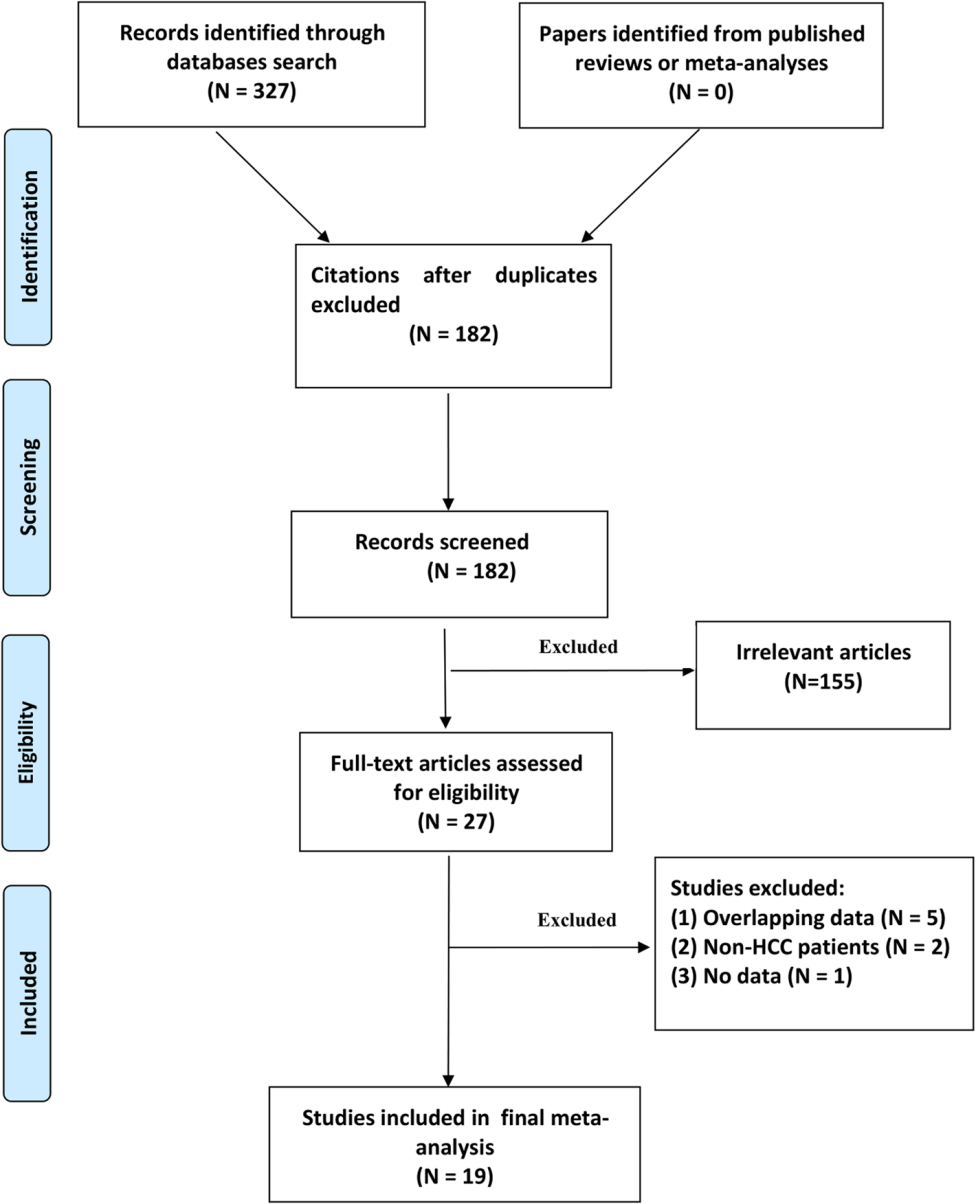
Flow chart of the study selection

A total of 182 studies were identified after the removal of duplicates from different databases. Twenty-seven articles passed title and abstract screening and underwent full-text review. Nineteen studies met the inclusion criteria and were included in the final analysis. A PRISMA flow chart showing the selection of studies for this meta-analysis is presented in Fig. 1. Eleven studies with 2138 cases and 4875 controls analyzed the *ALDH2* rs671 polymorphism [6–8, 14–21], while 12 studies including 1418 cases and 1701 controls assessed the *CYP2E1* rs2031920 polymorphism [11, 15–17, 21–28]. The quality score of the eligible studies ranged from 6 to 8 points. The characteristics of the included studies are summarized in Tables 1 and 2.

**Table 1:** Characteristics of the studies assessing *ALDH2* rs671 and HCC susceptibility

Author	Country	Year	Cases				Controls				HWE	Genotyping method	Virus infection		Quality score
			Total	GG	GA	AA	Total	GG	GA	AA			Cases	Controls	
Takeshita	Japan	2000	102	62	38	2	125	65	49	11	Yes	PCR-RFLP	8 with HBsAg (+); 71 with HCV antibody (+)	HBsAg (+), 0%; HCV antibody (+), 0%	7
Koide	Japan	2000	84	48	32	4	84	43	33	8	Yes	PCR-RFLP	HBsAg (+), 14.5%; anti-HCV (+), 81.9%	HBsAg (+), 0%; anti-HCV (+), 7.1%	6
Yu	China	2002	132	67	51	14	134	58	63	13	Yes	PCR	HBsAg (+), 67.9%; anti-HCV (+), 5.2%	HBsAg (+), 15.7%; anti-HCV (+), 6.0%	8
Munaka	Japan	2003	78	34	44 (GA + AA)		138	76	62 (GA + AA)		Yes	PCR	HBsAg (+), 17.9%; anti-HCV (+), 69.2%	HBsAg (+), 0.7%; anti-HCV (+), 7.3%	8
Kato	Japan	2003	94	75 (GG + GA)		19	133	127 (GG + GA)		6	Yes	PCR-RFLP	HBsAg (+), not reported; anti-HCV (+), 100%	HBsAg (+), not reported; anti-HCV (+), 0%	7
Sakamoto	Japan	2006	209	117	77	15	275	146	107	22	Yes	PCR-CTPP	HBsAg (+), 9.1%; anti-HCV (+), 85.6%	HBsAg (+), 2.2%; anti-HCV (+), 7.6%	8
Ding	China	2008	208	120	64	24	207	133	59	15	No	PCR-RFLP	HBsAg (+), 72.1%; anti-HCV (+), not reported	HBsAg (+), 22.2%; anti-HCV (+), not reported	8
Tomoda	Japan	2012	264	132	111	21	199	126	60	13	Yes	PCR	HBsAg (+), 0%; anti-HCV (+), 100%	HBsAg (+), 0%; anti-HCV (+), 100%	6
Abe	Japan	2015	67	51	16	0	67	62	5	0	Yes	PCR-CTPP	HBsAg (+), 0%; anti-HCV (+), 0%	HBsAg (+), 0%; anti-HCV (+), 0%	8
Liu	China	2016	600	303	248	49	3221	1617	1354	250	Yes	PCR	HBsAg (+), 100%; anti-HCV (+), 0%	HBsAg (+), 100%; anti-HCV (+),0%	7
Ye	China	2018	300	149	121	30	292	152	119	21	Yes	PCR-RFLP	HBsAg (+), 85.0%; anti-HCV (+), not reported	HBsAg (+), 10.3%; anti-HCV (+), not reported	8

For *ALDH2* rs671, the G allele is the wild-type allele

*CTPP* confronting two-pair primers, *HWE* Hardy-Weinberg equilibrium, *NOS* Newcastle Ottawa Scale, *PCR* polymerase chain reaction, *RFLP* restriction fragment length polymorphism

**Table 2:** Characteristics of the studies analyzing *CYP2E1* rs2031920 and HCC susceptibility

Author	Country	Year	Cases				Controls				HWE	Genotyping method	Virus infection		Quality score
			Total	CC	CT	TT	Total	CC	CT	TT			Cases	Controls	
Yu	China	1995	30	25	5	0	150	95	49	6	Yes	PCR-RFLP	HBsAg (+), 96.7%; anti-HCV (+), 16.7%	HBsAg (+), 49.3%; anti-HCV (+), 4.0%	7
Lee	Korea	1997	108	67	36	5	31	23	6	2	Yes	PCR-RFLP	HBsAg (+), not reported; anti-HCV (+), not reported	HBsAg (+), not reported; anti-HCV (+), not reported	8
Liu	China	2000	84	60	22	2	144	80	57	7	Yes	PCR-RFLP	HBsAg (+), not reported; anti-HCV (+), not reported	HBsAg (+), not reported; anti-HCV (+), not reported	7
Yu	China	2002	131	83	41	7	134	77	47	10	Yes	PCR-RFLP	HBsAg (+), 67.9%; anti-HCV (+), 5.2%	HBsAg (+), 15.7%; anti-HCV (+), 6.0%	8
Kato	Japan	2003	93	57	36 (CT + TT)		115	68	47 (CT + TT)		Yes	PCR-RFLP	HBsAg (+), not reported; anti-HCV (+), 100%	HBsAg (+), not reported; anti-HCV (+), 0%	7
Munaka	Japan	2003	77	45	32 (CT + TT)		138	89	49 (CT + TT)		Yes	PCR-RFLP	HBsAg (+), 17.9%; anti-HCV (+), 69.2%	HBsAg (+), 0.7%; anti-HCV (+), 7.3%	8
Meng	China	2003	21	1	19	1	50	36	14	0	Yes	PCR-RFLP	HBsAg (+), not reported; anti-HCV (+), not reported	HBsAg (+), not reported; anti-HCV (+), not reported	6
Jiang	China	2004	207	122	76	9	208	131	67	10	Yes	PCR-RFLP	HBsAg (+), not reported; anti-HCV (+), not reported	HBsAg (+), not reported; anti-HCV (+), not reported	6
Wu	China	2007	63	43	17	3	86	47	31	8	Yes	PCR-RFLP	HBsAg (+), not reported; anti-HCV (+), not reported	HBsAg (+), not reported; anti-HCV (+), not reported	8
Imaizumi	Japan	2009	209	127	73	9	256	160	83	13	Yes	PCR-RFLP	HBsAg (+) 9.1%; anti-HCV (+), 85.6%	HBsAg (+), 2.3%; anti-HCV (+), 7.8%	6
Di	China	2013	95	80	15	0	97	84	13	0	Yes	PCR-RFLP	HBsAg (+), not reported; anti-HCV (+), not reported	HBsAg (+), not reported; anti-HCV (+), not reported	8
Ye	China	2018	300	203	87	10	292	196	81	15	Yes	PCR-RFLP	HBsAg (+), 85.0%; anti-HCV (+), not reported	HBsAg (+), 10.3%; anti-HCV (+), not reported	8

For *CYP2E1* rs2031920, the C allele is the wild-type allele

*HWE* Hardy-Weinberg equilibrium, *NOS* Newcastle Ottawa Scale, *PCR-RFLP* polymerase chain reaction-restriction fragment length polymorphism

### Quantitative synthesis

**Fig. 2:**
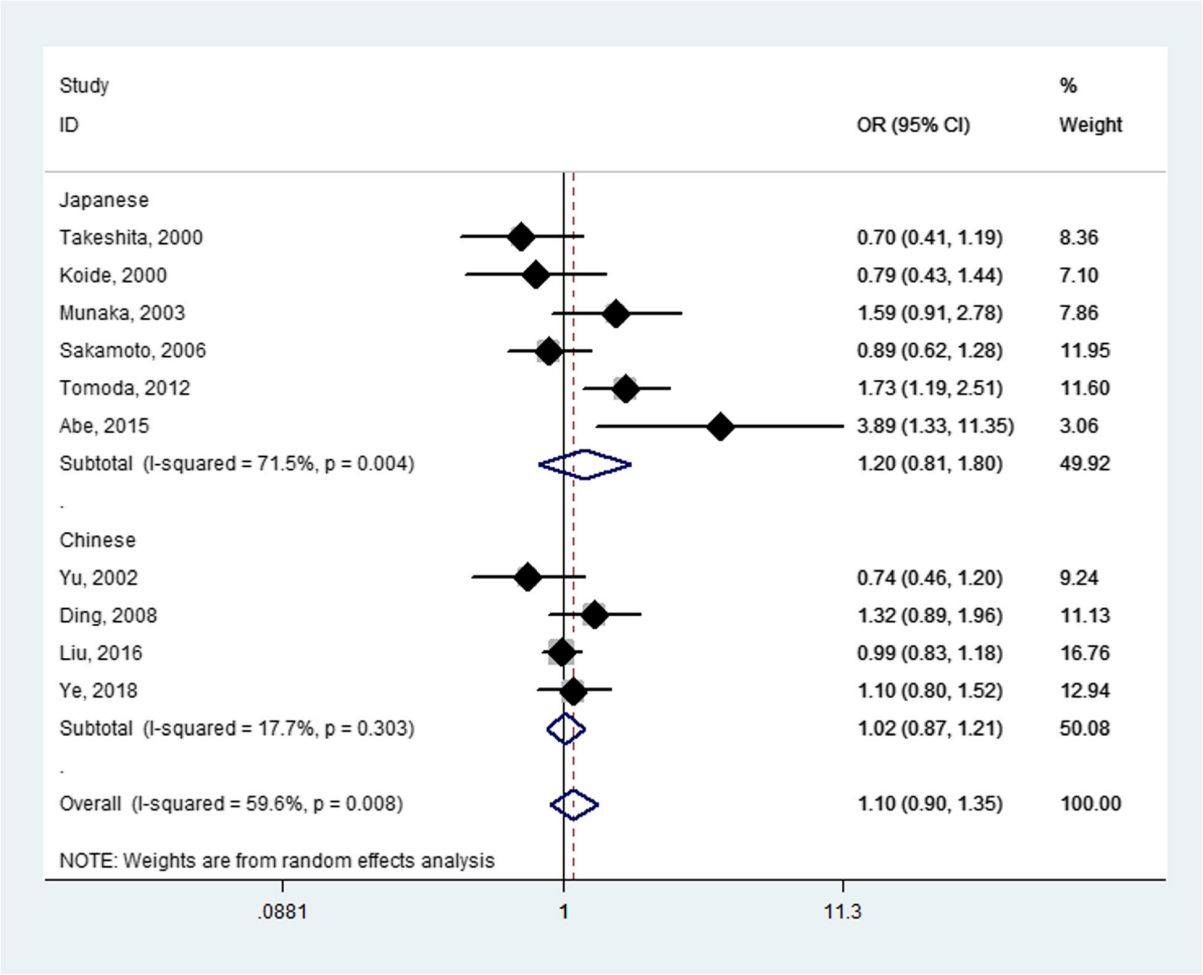
Forest plot for meta-analysis of the ALDH2 rs671 polymorphism and hepatocellular carcinoma susceptibility (AA + GA vs. GG)

The *ALDH2* rs671 polymorphism was evaluated in Chinese and Japanese populations. The overall meta-analysis did not suggest any association between *ALDH2* rs671 and HCC susceptibility for AA + GA genotype vs. GG genotype (OR = 1.10, *P* = 0.369), AA genotype vs. GA + GG genotype (OR = 1.19, *P* = 0.357), AA genotype vs. GG genotype (OR = 1.08, *P* = 0.509), and GA genotype vs. GG genotype (OR = 1.06, *P* = 0.569; Fig. 2 and Table 3). The sensitivity analysis revealed that omitting the study by Ding et al. which deviated from Hardy-Weinberg equilibrium had no effect on the overall outcome of disease risk [18] (Table 3). Through subgroup analyses by country, no significant associations were found in Chinese or Japanese (Fig. 2 and Table 3). A subgroup analysis by source of controls (population-based and hospital-based) also did not identify any association (Table 3).

**Table 3:** Meta-analysis results for *ALDH2* rs671

Genotype and subgroup	Number of studies	Test of association		Test of heterogeneity		*P* of Egger’s test
		OR (95% CI)	*Z* (*P* value)	*I*^2^ (%)	*P*_het_	
AA + GA vs. GG						
Overall	10	1.10 (0.90–1.35)	0.369	59.6	0.008	0.430
HWE (yes)	9	1.08 (0.86–1.35)	0.527	62.0	0.007	
HWE (no)	1	1.32 (0.89–1.96)	0.171	NA	NA	
Chinese	4	1.03 (0.87–1.21)	0.770	17.7	0.303	
Japanese	6	1.21 (0.81–1.80)	0.362	71.5	0.004	
Population-based studies	4	0.98 (0.80–1.20)	0.859	24.1	0.267	
Hospital-based studies	6	1.24 (0.88–1.75)	0.210	68.9	0.007	
AA vs. GA + GG						
Overall	9	1.19 (0.82–1.73)	0.357	58.2	0.014	0.866
HWE (yes)	8	1.13 (0.75–1.71)	0.562	61.2	0.012	
HWE (no)	1	1.67 (0.85–3.28)	0.137	NA	NA	
Chinese	4	1.19 (0.93–1.52)	0.165	0	0.589	
Japanese	5	1.00 (0.41–2.42)	1.000	76.8	0.002	
Population-based studies	4	1.10 (0.82–1.49)	0.521	8.6	0.350	
Hospital-based studies	5	1.26 (0.62–2.56)	0.526	73.7	0.004	
AA vs. GG						0.289
Overall	8	1.08 (0.87–1.34)	0.509	37.1	0.133	
HWE (yes)	7	1.02 (0.81–1.28)	0.892	33.6	0.171	
HWE (no)	1	1.77 (0.89–3.54)	0.104	NA	NA	
Chinese	4	1.18 (0.92–1.52)	0.191	0	0.452	
Japanese	4	0.82 (0.54–1.26)	0.373	58.0	0.067	
Population-based studies	4	1.08 (0.74–1.57)	0.690	24.7	0.263	
Hospital-based studies	4	1.00 (0.54–1.85)	0.992	58.1	0.067	
GA vs. GG						
Overall	9	1.06 (0.86–1.31)	0.569	54.2	0.026	0.464
HWE (yes)	8	1.05 (0.83–1.32)	0.697	58.7	0.018	
HWE (no)	1	1.20 (0.78–1.85)	0.403	NA	NA	
Chinese	4	0.99 (0.85–1.14)	0.833	0	0.452	
Japanese	5	1.21 (0.78–1.89)	0.395	70.0	0.010	
Population-based studies	4	0.97 (0.83–1.13)	0.669	0	0.452	
Hospital-based studies	5	1.22 (0.83–1.78)	0.305	69.2	0.011	

For *ALDH2* rs671, the G allele is the wild-type allele

*CI* confidence interval, *NA* not applicable, *OR* odds ratio

**Fig. 3:**
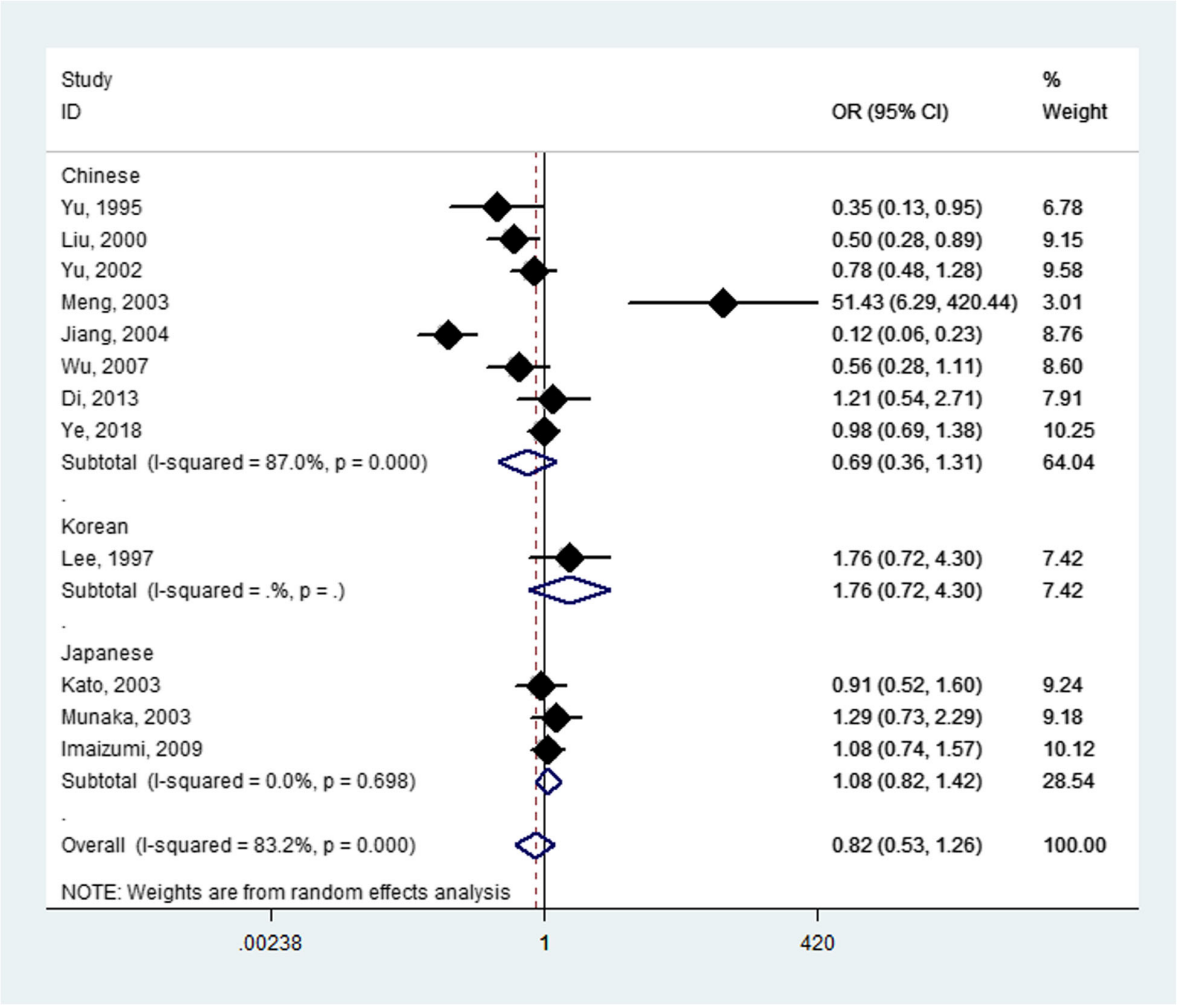
Forest plot for meta-analysis of the CYP2E1 rs2031920 polymorphism and hepatocellular carcinoma susceptibility (CT + TT vs. CC)

The *CYP2E1* rs2031920 polymorphism was assessed in Chinese, Japanese, and Korean populations. There was no association between the polymorphism and HCC susceptibility when combining the results from all eligible studies (OR = 0.82, *P* = 0.358 for TT + CT vs. CC; OR = 0.72, *P* = 0.096 for TT vs. CT + CC; OR = 0.54, *P* = 0.079 for TT vs. CC; OR = 0.97, *P* = 0.886 for CT vs. CC; Fig. 3 and Table 4). All studies conformed to Hardy-Weinberg equilibrium. In the subgroup analysis by country, we did not find any association of *CYP2E1* rs2031920 with HCC susceptibility in Chinese (Fig. 3 and Table 4), Japanese, and Koreans. When the included studies were subgrouped according to the source of controls, the analyses did not show any statistically significant results (Table 4).

**Table 4:** Meta-analysis results for *CYP2E1* rs2031920

Genotype and subgroup	Number of studies	Test of association		Test of heterogeneity		*P* of Egger’s test
		OR (95% CI)	*Z* (*P* value)	*I*^2^ (%)	*P*_het_	
CT + TT vs. CC						
Overall	12	0.82 (0.53–1.26)	0.358	83.2	0.000	0.843
Chinese	8	0.69 (0.37–1.31)	0.259	87.0	0.000	
Japanese	3	1.08 (0.82–1.42)	0.587	0	0.698	
Korean	1	1.76 (0.72–4.30)	0.215	NA	NA	
Population-based studies	2	0.31 (0.05–1.98)	0.214	95.2	0.000	
Hospital-based studies	10	0.97 (0.68–1.39)	0.871	68.7	0.001	
TT vs. CT + CC						
Overall	9	0.72 (0.49–1.06)	0.096	0	0.921	0.714
Chinese	7	0.69 (0.44–1.08)	0.107	0	0.803	
Japanese	1	0.84 (0.35–2.01)	0.697	NA	NA	
Korean	1	0.70 (0.13–3.82)	0.684	NA	NA	
Population-based studies	2	0.80 (0.41–1.57)	0.520	0	0.717	
Hospital-based studies	7	0.68 (0.43–1.10)	0.115	0	0.818	
TT vs. CC						
Overall	9	0.54 (0.27–1.08)	0.079	59.0	0.012	0.523
Chinese	7	0.47 (0.19–1.16)	0.102	65.1	0.009	
Japanese	1	0.87 (0.36–2.11)	0.761	NA	NA	
Korean	1	0.86 (0.16–4.73)	0.861	NA	NA	
Population-based studies	2	0.25 (0.04–1.67)	0.153	84.9	0.010	
Hospital-based studies	7	0.70 (0.37–1.31)	0.266	27.2	0.221	
CT vs. CC						
Overall	9	0.97 (0.67–1.41)	0.886	70.6	0.001	0.595
Chinese	7	0.89 (0.56–1.42)	0.624	75.2	0.000	
Japanese	1	1.11 (0.75–1.64)	0.607	NA	NA	
Korean	1	2.06 (0.77–5.52)	0.151	NA	NA	
Population-based studies	2	1.03 (0.69–1.52)	0.899	31.4	0.227	
Hospital-based studies	7	1.00 (0.59–1.68)	0.994	76.6	0.000	

For *CYP2E1* rs2031920, the C allele is the wild-type allele

*CI* confidence interval, *NA* not applicable, *OR* odds ratio

### Heterogeneity and meta-regression

Significant heterogeneity was found among the studies evaluating rs671 and rs2031920 (Tables 3 and 4). We performed a meta-regression analysis to explore the potential modifiers contributing to the heterogeneity between the studies that assessed rs671. Year of publication, country, source of controls, and sample size were considered. However, the results showed that these factors were not the sources of heterogeneity (*P* = 0.101 for year of publication; *P* = 0.606 for country; *P* = 0.366 for source of controls; *P* = 0.212 for sample size). The meta-regression results for rs2031920 were similar. Next, we conducted the Galbraith plot and accordingly singled out the studies of Tomoda et al. [19] and Abe et al. [20] as the main sources of heterogeneity for rs671 (graph not shown). Removing these studies decreased heterogeneity (*P*_het_ = 0.247, *I*^2^ = 22.9%), without significantly influencing the pooled ORs. For rs2031920, removing the studies by Meng et al. [24] and Jiang et al. [25] significantly reduced between-study heterogeneity (*P*_het_ = 0.096, *I*^2^ = 39.4%) but did not alter the corresponding pooled ORs.

### Cumulative meta-analysis and publication bias

**Fig. 4:**
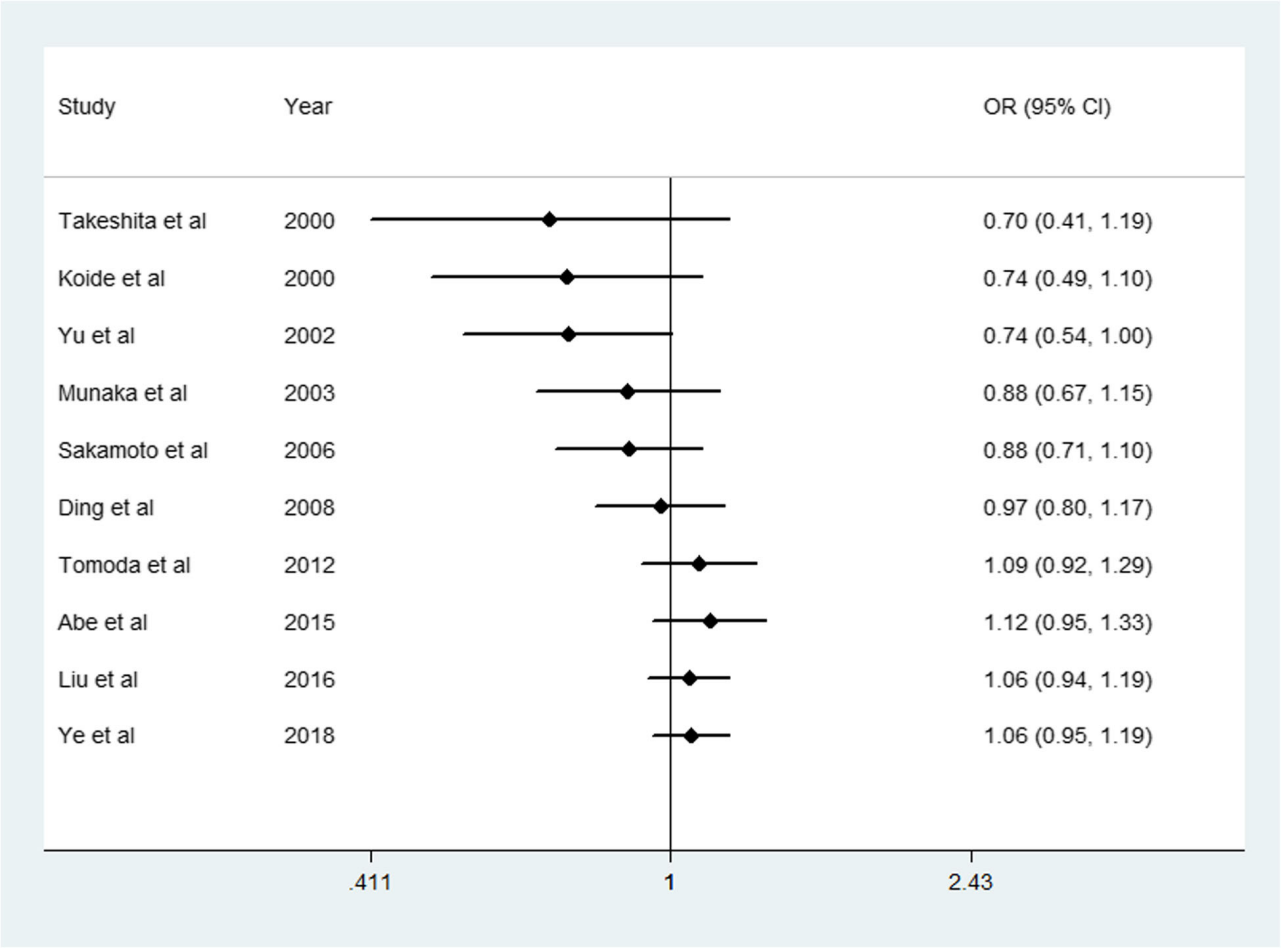
Cumulative meta-analysis of the ALDH2 rs671 polymorphism and hepatocellular carcinoma susceptibility (AA + GA vs. GG)

**Fig. 5:**
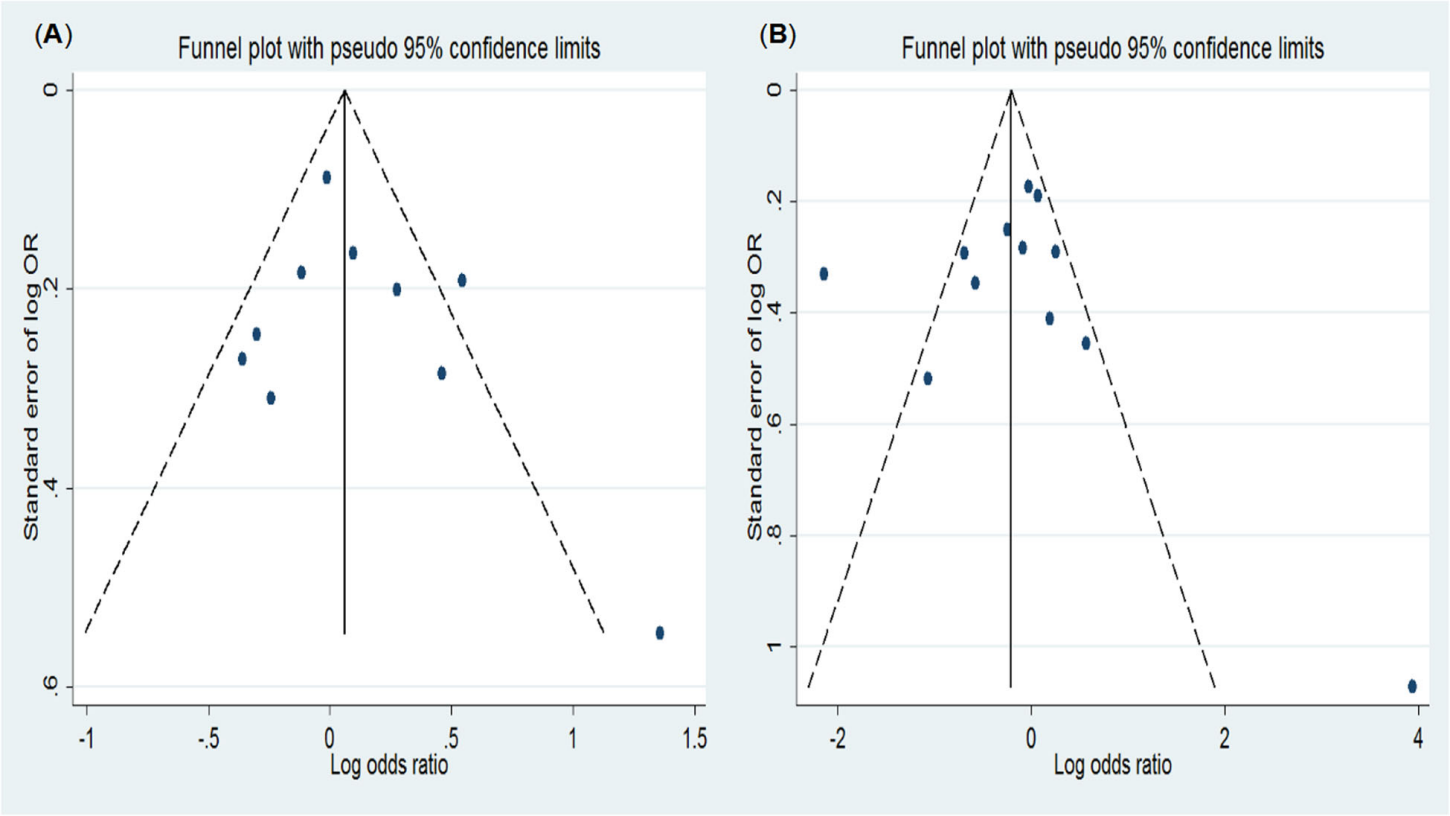
Evaluation of publication bias. **a** Funnel plot analysis to detect publication bias for the ALDH2 rs671 polymorphism (AA + GA vs. GG). **b** Funnel plot analysis to detect publication bias for the CYP2E1 rs2031920 polymorphism (CT + TT vs. CC)

We performed a cumulative meta-analysis to explore the trend in the effect sizes. The calculation showed a lack of association between *ALDH2* rs671 and HCC susceptibility (Fig. 4). The results for *CYP2E1* rs2031920 were similar (not shown). To evaluate the publication bias, a funnel plot of the logarithm of effect size (logOR) against the precision for each study was generated (Fig. 5). There was no evidence of publication bias using Egger’s test (Tables 3 and 4).

## Discussion

HCC is the major cause of cancer mortality in some parts of Asia. The poor prognosis of HCC accentuates the need to develop novel genetic markers and therapeutic approaches. Over the past two decades, the relationship of *ALDH2* rs671 and *CYP2E1* rs2031920 with HCC susceptibility has been extensively studied among East Asian populations, but there are inconsistencies in the results. In the present study, we reviewed the available literature and performed a meta-analysis regarding these associations. Our results showed no significant effect of *ALDH2* rs671 and *CYP2E1* rs2031920 on susceptibility to HCC in East Asians under various genetic models.

This is the largest and most comprehensive meta-analysis on the relationship of *ALDH2* rs671 and *CYP2E1* rs2031920 with HCC susceptibility in East Asians. The evaluation of *ALDH2* rs671 was based on 11 studies with 2138 cases and 4875 controls, whereas 12 studies including 1418 cases and 1701 controls were reviewed for *CYP2E1* rs2031920. In addition to the overall meta-analyses, we performed subgroup analyses by country (Chinese, Japanese, and Koreans), Hardy-Weinberg equilibrium status, and source of controls. Moreover, we conducted a cumulative meta-analysis to see how the evidence had shifted over time. These efforts did not identify any association of *ALDH2* rs671 and *CYP2E1* rs2031920 with HCC susceptibility. Our findings were supported by most of the included studies. Among the 11 studies evaluating *ALDH2* rs671, 8 reported no association with HCC, including the study by Liu et al. which had the largest sample size (600 cases and 3221 controls) [7]. Concerning *CYP2E1* rs2031920, 9 studies did not observe any association. Yu et al. evaluated the association between *CYP2E1* rs2031920 and HCC susceptibility in a Chinese population for the first time; they found no association for the *CYP2E1* polymorphism [11]. Null association between *CYP2E1* rs2031920 and HCC susceptibility was also reported in several Japanese and Korean studies [16, 17, 22, 27]. The findings of the published case-control studies, together with the outcomes from this meta-analysis, suggested that *ALDH2* rs671 and *CYP2E1* rs2031920 were unlikely to be major contributors to HCC susceptibility in East Asian populations.

There was significant heterogeneity between the included studies. For exploring the potential modifiers contributing to heterogeneity, we conducted a meta-regression analysis. We showed that year of publication, country, source of controls, and sample size were not the main contributors to heterogeneity. We did not take into account other factors such as sex ratio, HBV/HCV status, and drinking habits, because not all studies reported the information. It was suggested that meta-regression was not always effective in explaining between-study heterogeneity [29]. In addition to meta-regression, we conducted the Galbraith plot to explore heterogeneity, finding that the studies of Tomoda et al. [19] and Abe et al. [20] were the sources of heterogeneity for *ALDH2* rs671. When these studies were omitted from the overall meta-analysis, the heterogeneity dropped down to 22.9% (*P*_het_ = 0.247), without significantly affecting the pooled ORs. Concerning *CYP2E1* rs2031920, Galbraith’s test showed that the studies of Meng et al. [24] and Jiang et al. [25] were the main contributors to heterogeneity; removing them did not alter the overall estimation. Thus, we ensured that the meta-analytic results were robust.

A previous meta-analysis by Zhou et al. reported no association between *ALDH2* rs671 and the risk of HCC in East Asians with a total of 1231 cases and 1849 controls [30]. Using a larger sample size (2138 cases and 4875 controls), our study confirmed their findings and provided more information through subgroup analysis and cumulative meta-analysis. In addition, we explored the source of heterogeneity, but Zhou et al. did not perform any analyses for it [30]. For *CYP2E1* rs2031920, our results contrasted with those of the meta-analysis by Tian et al. which reported an association between rs2031920 and HCC susceptibility in East Asians [31]. Tian and colleagues’ results may be false positive, because they included studies deviating from Hardy-Weinberg equilibrium and pooled overlapping data from the same research group. Two other meta-analyses evaluated the association of rs2031920 with HCC susceptibility using Asian, Caucasian, and Hispanic populations together, but did not find any significant association [32, 33].

Our meta-analysis suggested a lack of association between *ALDH2* rs671 and HCC susceptibility, but we could not exclude the possibility that an interaction between *ALDH2* rs671 and alcohol drinking may have a role in the development of HCC. Abe et al. found that the profile of alcohol consumption and *ALDH2* rs671 had a close relation, and *ALDH2* rs671 and the consumptive period affected HCC development in patients with alcoholic liver cirrhosis [20]. In addition, the study by Liu et al. suggested that the association between *ALDH2* rs671 and HCC might be significantly mediated by habitual alcohol consumption [7]. However, a principal limitation of these studies was the definition of alcohol drinking, which may cause selection bias. Another limitation was that viral infection was not taken into account. It is known that chronic HBV or HCV infection is common in the Asian continent; adjustment for viral infection may be necessary to clarify whether potential interactions between *ALDH2* rs671 and alcohol drinking contribute to HCC susceptibility.

Some limitations of our meta-analysis should be considered. First, the eligible studies in our meta-analysis were mainly from Chinese and Japanese. There was only one study from Koreans [22]. Chronic infection with HBV is the predominant risk factor for HCC in China and Korea, while chronic HCV infection is the risk factor for HCC in Japan [34]. A subgroup analysis was performed to evaluate the association of these polymorphisms with HCC in different countries. Second, most of the included studies were hospital based. The controls may not reflect the representative element of the source population. Third, although Egger’s test and funnel plots did not suggest publication bias, selection bias might have occurred, because we included only studies written in English and Chinese. Fourth, owing to the insufficient information, we did not perform a subgroup analysis by gender.

In conclusion, the results of our meta-analysis suggest that *ALDH2* rs671 and *CYP2E1* rs2031920 are not associated with susceptibility to HCC in East Asians. Further, well-designed and population-based studies are needed to evaluate the potential interaction between these polymorphisms and alcohol drinking in HCC susceptibility.

## Supplementary information

**Additional file 1:** **Table S1.** Database search strategy.

## Data Availability

All data generated or analyzed during this study are included in this published article.

## Abbreviations

ACE: Acetaldehyde
ALDH2: Aldehyde dehydrogenase 2
CI: Confidence interval
CYP2E1: Cytochrome p450 2E1
HBV: Hepatitis B virus
HCC: Hepatocellular carcinoma
HCV: Hepatitis C virus
NOS: Newcastle Ottawa Scale
OR: Odds ratio
PRISMA: Preferred Reporting Items for Systematic Reviews and Meta-Analyses
