# Supplementary material for: Relationship of ALDH2 rs671 and CYP2E1 rs2031920 with hepatocellular carcinoma susceptibility in East Asians: a meta-analysis
Source: World J Surg Oncol. 2020 Jan 27;18:21. doi: 10.1186/s12957-020-1796-0 (PMC6986079; doi:10.1186/s12957-020-1796-0)
Supplement: Supplementary file 1 — Additional file 1: Table S1. Database search strategy. [file 12957_2020_1796_MOESM1_ESM.docx]

Supplementary table 1 Database search strategy.

| **Database** | Medline, Pubmed, Scopus, Embase and China Academic Journals databases |
| --- | --- |
| **Date** | From inception to July 8, 2019 |
| **Eligibility criteria** | Case-control studies that analyzed the relation of *ALDH2* rs671 and *CYP2E1* rs2031920 polymorphisms with susceptibility to HCC, published in Chinese and English |
| **Keywords** | hepatocellular carcinoma, liver cancer, aldehyde dehydrogenase 2, ALDH2, cytochrome p450 2E1, CYP2E1, polymorphism, genetic variant, susceptibility and development |
